# Isolated greater trochanter fracture may impose a comparable risk on older patients’ survival as a conventional hip fracture: a population-wide cohort study

**DOI:** 10.1186/s12891-022-05336-3

**Published:** 2022-04-27

**Authors:** Pärt Prommik, Kaspar Tootsi, Karin Veske, Eiki Strauss, Toomas Saluse, Helgi Kolk, Aare Märtson

**Affiliations:** 1 Department of Traumatology and Orthopaedics, University of Tartu, L. Puusepa 8, 50406 Tartu, Estonia; 2 Traumatology and Orthopaedics Clinic, Tartu University Hospital, L. Puusepa 8, 50406 Tartu, Estonia

**Keywords:** Isolated greater trochanter fracture, Hip fracture, Survival, Mortality risk, Acute management, Post-acute management

## Abstract

**Background:**

Isolated greater trochanter fracture (IGT) and conventional hip fracture (HF) affect the same anatomical area but are usually researched separately. HF is associated with high mortality, and its management is well established. In contrast, IGT’s effect on mortality is unknown, and its best management strategies are unclear. This study aims to compare these patient populations, their acute- and post-acute care, physical and occupational therapy use, and up to three-year mortality.

**Methods:**

This retrospective cohort study is based on population-wide data of Estonia, where routine IGT management is non-operative and includes immediate weight-bearing as tolerated. The study included patients aged ≥ 50 years with a validated index HF or IGT diagnosis between 2009–2017. The fracture populations’ acute- and post-acute care, one-year physical and occupational therapy use and three-year mortality were compared.

**Results:**

A total of 0.4% (50/11,541) of included patients had an IGT. The baseline characteristics of the fracture cohorts showed a close resemblance, but the IGT patients received substantially less care. Adjusted analyses showed that the IGT patients’ acute care was 4.5 days [3.4; 5.3] shorter they had 39.2 percentage points [25.5; 52.8] lower probability for receiving post-acute care, and they had 50 percentage points [5.5: 36]] lower probability for receiving physical and occupational therapy. The IGT and HF patients’ mortality rates were comparable, being 4% and 9% for one month, 28% and 31% for one year, and 46% and 49% for three years, respectively. Crude and adjusted analyses could not find significant differences in their three-year mortality, showing a *p*-value of 0.6 and a hazard ratio of 0.9 [0.6; 1.3] for the IGT patients, retrospectively.

**Conclusions:**

Despite IGT being a relatively minor injury, the evidence from this study suggests that it may impose a comparable risk on older patients’ survival, as does HF due to the close resemblance of the two fracture populations. Therefore, IGT in older patients may signify an underlying need for broad-based medical attention, ensuring need-based, ongoing, coordinated care.

**Trial registration:**

Retrospectively registered.

## Background

Ageing populations are leading to an enormous increase in the incidence of fragility fractures. To disrupt the status quo of the fragility fracture crisis, several leading organisations have jointly published a “Global Call to Action,” which highlights the urgent need to improve fracture patients’ care [1]. Of the different types of fragility fractures, hip fracture (HF) is associated with some of the most serious consequences: high mortality, limited functional recovery, and the imposition of a massive burden on patients, their families, health systems, and society in general [2–6]. HF management is well established, being both time-critical and broad-based. HF management’s three fundamental care pillars are acute multidisciplinary care, ongoing coordinated rehabilitation, and rapid secondary prevention [1].

Isolated greater trochanter fracture (IGT) is a rare musculoskeletal trauma. Although it is not considered as a fragility fracture, it has many similarities with conventional HF: it affects the same anatomical area, older IGT patients also have underlying osteoporosis, and is often a result of a low energy trauma [7–11]. Despite the similarities, these fracture populations are usually researched separately, IGT is less studied, and its best management strategies are unknown [8, 10, 12]. IGT management varies from operative to non-operative, depending on the length of intertrochanteric extension [9, 11–15]. In Estonia, routine IGT management is non-operative: the aim is to keep patients ambulatory using assistive devices and weight-bearing as tolerated.

To date, no studies have compared these fracture populations and their ongoing management. The effect of IGT on patients’ survival is also unknown. Only two small studies have reported IGT mortality, and their findings are conflicting. One reported 11.9% one-month mortality, and the second showed that none of 30 included patients died within a year [9, 11]. For these reasons, this study aims to compare these fracture populations, their acute and post-acute care, physical and occupational therapy use, and up to three-year mortality.

## Methods

A nationwide and population-based retrospective cohort study was conducted using data from the database of the Estonian Health Insurance Fund (EHIF), which is a unified social insurance system, covering 94%-95% of the Estonian population [16, 17]. EHIF collects data from billings from hospitals (medical data), the Estonian Population Register (demographics) and the Estonian Causes of Death Registry (survival status). Billing data covers service use in all care settings, including inpatient (acute care, nursing care, rehabilitation care), day care, and outpatient care (ambulatory specialist care, nursing care rehabilitation care, primary care) [17]. This study included patients aged 50 and over with an index IGT or HF diagnosis between 1 January 2009 and 30 September 2017. The initial fracture diagnoses were based on the 10^th^ revision of the International Classification of Diseases codes (ICD-10) S72.0–2. The fracture diagnoses were later validated. The validation excluded patients with a pelvic, a periprosthetic, a secondary HF, a pathologic IGT, an isolated acetabular and lesser trochanter fracture, and an IGT supported by unconvincing radiological evidence (e.g., poor image quality). Secondary HFs are associated with increased mortality risk, and pathologic IGTs may lead to an overestimation of the fracture’s effect on mortality [18].

Multiple EHIF datasets were retrieved, including patient demographics, ICD-10 codes for up to four years pre-fracture, the Nordic Medico-Statistical Committee’s Classification of Surgical Procedures (NCSP) codes for up to three months post-fracture, all prior S72.0–2 codes, and medical claims data of acute and post-acute care episodes up to one-year post-fracture. The data also included the amount of physical and occupational therapy used during each care episode. Observation time for the primary outcome measure – survival – was censored for both comparison groups three years from fracture incidence. All patients had a complete follow-up for survival. Secondary outcome measures were acute hospital length of stay and the probability of receiving post-acute care and physiotherapy and occupational therapy, and their total amount.

### Data validation

**Fig. 1:**
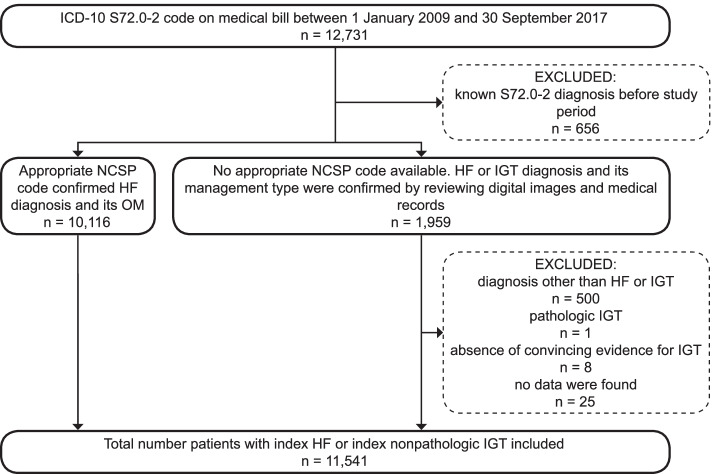
Flowchart showing the validation of HF and IGT diagnoses. HF – conventional hip fracture, ICD-10 – International Classification of Diseases, 10^th^ Revision, IGT – isolated greater trochanter fracture, NCSP—Nordic-Medico-Statistical Committee’s Classification of Surgical Procedures, OM – operative management

Multiple validation steps were used to confirm HF and IGT diagnoses (Fig. 1). First, HF diagnoses were validated by a logic check: patients with an appropriate NCSP code were considered to have HF fractures. The following NCSP codes were considered as appropriate in the logic check: total hip arthroplasty (NFB20, NFB30, NFB40, NFB99), hemiarthroplasty (NFB00-9; NFB10-9), screws (NFJ70-3), sliding hip screw (NFJ60-3, NFJ80-3) and intramedullary nail (NFJ50-3) [19]. If NCSP codes were not available, patients’ digital imaging and medical records were reviewed to confirm the remaining patients’ HF diagnoses or IGT diagnoses. Two national databases were used for reviewing digital imaging and medical records: the Foundation of Estonian PACS (an image archiving and communication system database) and the Estonian National Health Information System (https://ap.digilugu.ee/arstiportaal). Uploading patients’ medical data to these databases has been mandatory for EHIF-funded hospitals since 2010 in the case of medical records and since 2014 for digital images. Voluntary uploading took place before these years. Digital images were reviewed by two experienced musculoskeletal radiologists and an orthopaedic surgeon to confirm fracture diagnosis and its management and to rule out the intertrochanteric extension or a pathologic IGT. A geriatrician reviewed medical records.

### Calculation of comorbidities

The Charlson Comorbidity Index (CCI) score was used for comorbidity assessment as this is validated on an HF population [20–22]. CCI was calculated using coding algorithms for ICD-10 codes [20] and updated weights [23]. A restriction was applied to improve the validity of comorbidity assessment: only those ICD-10 codes which appeared at least twice and at least seven days apart were included [21, 24]. The presence of comorbid conditions was subtracted from the CCI [20, 25].

### Statistical analysis

Statistical analyses were performed in R 4.0.4 (R Core Team, 2017), using the following packages: *survival,* and *survminer* for crude survival analyses. Bayesian R packages were used for adjusted analyses: *brms* [26] for negative binomial, lognormal, logistic regression, and *rstanarm* [27] for survival modelling. Comorbidity scores were calculated using a validated MS Excel-based calculator [28]. Adobe Illustrator or Adobe InDesign (versions CC, Adobe Systems, San Jose, CA) were used for creating or finalising figures.

Continuous and ordinal variables are shown as a ‘median (25^th^-75^th^ percentile)’, and categorical variables as proportions or probabilities. Two variables were grouped into categorical counterparts: 10-year subgroups for age and CCI as 0, 1–2, and ≥ 3. Continuous variables were compared using the Mann Whitney U-test. The Pearson chi-squared test was used for proportional comparisons. A Kaplan–Meier analysis was conducted up to three years from fracture diagnosis. The log-rank test was used to compare crude cumulative mortality. Statistical significance was defined as *p* < 0.05, and all tests were two-sided.

The regression models were adjusted for age, sex, and CCI. Negative binomial regression was used to compare the acute hospital length of stay. Logistic regression was performed using the Bernoulli likelihood to estimate the probability of receiving post-acute care and physical and occupational therapy. The positive hours of these therapies were analysed using lognormal likelihood. The survival regression model based on M-splines baseline hazard using 8 degrees of freedom, and the ‘age’ variable was specified as a time-varying coefficient, as it violated the assumption of proportional hazards [29]. All models were run with default weakly informative priors, and point estimates [mean days, probabilities, or hazard ratios (HR)] were given with 95% credible intervals (CI) as ‘[lower; upper].’

## Results

A total of 11,541 patients were included in the study, of which 0.4% (50) had IGT (Fig. 1). The IGT patients’ diagnoses were based on digital imaging: conventional radiography – 50% (25/50), computed tomography – 14% (7/50), and both methods – 24% (18/50). Only three IGT patients received bone density scanning. One received it just after the diagnosis; the remaining two received it 11 and 17 months later. The scanning showed osteoporosis for two of these patients. The remaining patient’s finding was normal. The baseline characteristics of these fracture populations showed a close resemblance in median age, sex, and CCI distributions (Table 1). Despite the similarities, the IGT patients received significantly less acute and post-acute care and physical and occupational therapy.

**Table 1:** Baseline characteristics of hip and isolated greater trochanter fracture patients

	Total *n* = 11,541	Hip fracture *n* = 11,491	Isolated greater trochanter fracture *n* = 50	*P*-value
Age	81 (73–87)	81 (73–87)	78 (65–87)	0.4
Age group				
50–59	801 (6.9)	791 (6.9)	10 (20.0)	0.006
60–69	1,411 (12.2)	1,406 (12.2)	5 (10.0)	
70–79	2,954 (25.6)	2,943 (25.6)	11 (22.0)	
80–89	4,881 (42.3)	4,865 (42.3)	16 (32.0)	
90 +	1,494 (12.9)	1,486 (12.9)	8 (16.0)	
Females	8,278 (71.7)	8,245 (71.8)	33 (66.0)	0.4
Fracture type				< 0.001
Femoral neck	5,883 (51.0)	5,883 (51.2)	0 (0.0)	
Pertrochanteric	4,953 (42.9)	4,953 (43.1)	0 (0.0)	
Subtrochanteric	655 (5.7)	655 (5.7)	0 (0.0)	
Isolated greater trochanter fracture	50 (0.4)	0 (0.0)	50 (100)	
Charlson Comorbidity Index				
0	4,510 (39.1)	4,495 (39.1)	15 (30.0)	0.4
1–2	4,147 (35.9)	4,127 (35.9)	20 (40.0)	
≥ 3	2,884 (25.0)	2,869 (25.0)	15 (30.0)	
Comorbidities				
Myocardial infarction	802 (6.9)	796 (6.9)	6 (12.0)	0.2
Congestive heart failure	5,052 (43.8)	5,025 (43.7)	27 (54.0)	0.14
Peripheral vascular disease	1,207 (10.5)	1,197 (10.4)	10 (20.0)	0.03
Cerebrovascular disease	2,484 (21.5)	2,477 (21.6)	7 (14.0)	0.2
Dementia	1,112 (9.6)	1,106 (9.6)	6 (12.0)	0.6
Chronic pulmonary disease	1,248 (10.8)	1,243 (10.8)	5 (10.0)	0.9
Rheumatic disease	384 (3.3)	383 (3.3)	1 (2.0)	0.6
Peptic ulcer disease	546 (4.7)	542 (4.7)	4 (8.0)	0.3
Mild liver disease	174 (1.5)	174 (1.5)	0 (0.0)	0.4
Diabetes without chronic complication	1,251 (10.8)	1,242 (10.8)	9 (18.0)	0.1
Diabetes with chronic complication	683 (5.9)	678 (5.9)	5 (10.0)	0.2
Hemi- or paraplegia	534 (4.6)	530 (4.6)	4 (8.0)	0.3
Renal disease moderate/severe	469 (4.1)	465 (4.0)	4 (8.0)	0.2
Any malignancy	1,184 (10.3)	1,179 (10.3)	5 (10.0)	> 0.9
Moderate/severe liver disease	36 (0.3)	36 (0.3)	0 (0.0)	0.7
Metastatic solid tumor	42 (0.4)	42 (0.4)	0 (0.0)	0.7
AIDS/HIV	1 (0.0)	1 (0.0)	0 (0.0)	0.9

Continuous variables are shown as median (25^th^-75^th^ percentile) and proportions as n (%)

*P*-values are based on the Mann Whitney U-test for continuous variables and the Pearson’s chi-squared test for categorical variables

The median acute length of stay for the IGT patients was 7 days shorter than for those with an HF diagnosis, and 54% of the IGT patients (27/50) received no post-acute care during the succeeding year from the diagnosis (Table 2). After adjusting for age, sex, and CCI, the IGT patients’ acute care was 4.5 days [3.4; 5.3] shorter, and they had 39.2 percentage points [25.5; 52.8] lower probability for receiving post-acute care. The IGT patients receiving post-acute care were more likely admitted to ambulatory or inpatient care (Table 2).

**Table 2:** Received acute and post-acute care of hip and isolated greater trochanter fracture patients

	Total *n* = 11,541	Hip fracture *n* = 11,491	Isolated greater trochanter fracture *n* = 50	*P*-value
Operative fracture management	10,442 (90.5)	10,442 (90.9)	0 (0.0)	< 0.001
Acute length of stay in days	8 (5–11)	8 (5–11)	1 (1–6)	< 0.001
Post-acute length of stay in days	15 (0–33)	15 (0–33)	0 (0–0)	< 0.001
Type of post-acute care				
Ambulatory	1,760 (15.2)	1,750 (15.2)	10 (20.0)	< 0.001
Combined	4,373 (37.9)	4,369 (38.0)	4 (8.0)	
Community	56 (0.5)	55 (0.5)	1 (2.0)	
Inpatient	3,763 (32.6)	3,755 (32.7)	8 (16.0)	
No post acute care	1,589 (13.8)	1,562 (13.6)	27 (54.0)	

Continuous variables are shown as median (25^th^-75^th^ percentile) and proportions as n (%)

*P*-values are based on the Mann Whitney U-test for continuous variables and the Pearson’s chi-squared test for categorical variables

During the first year from the diagnosis, IGT patients had worse chances of receiving physical and occupational therapy as these were received by 38% (19/50) of IGT and 87% (10,293/11491) of HF patients. After adjusting for age, sex, and CCI, the IGT patients had 50% percentage points [36; 63] lower chances of receiving these therapies. When comparing the amount of physical and occupational therapy of those included in rehabilitation, the IGT and HF patients received a total of 8.0 h (2.8–23.5) and 20.0 h (5.5–36.0), respectively. When adjusting for age, sex and CCI, the IGT patients received 16.1 h less [4; 24.5] physical and occupational therapy.

**Fig. 2:**
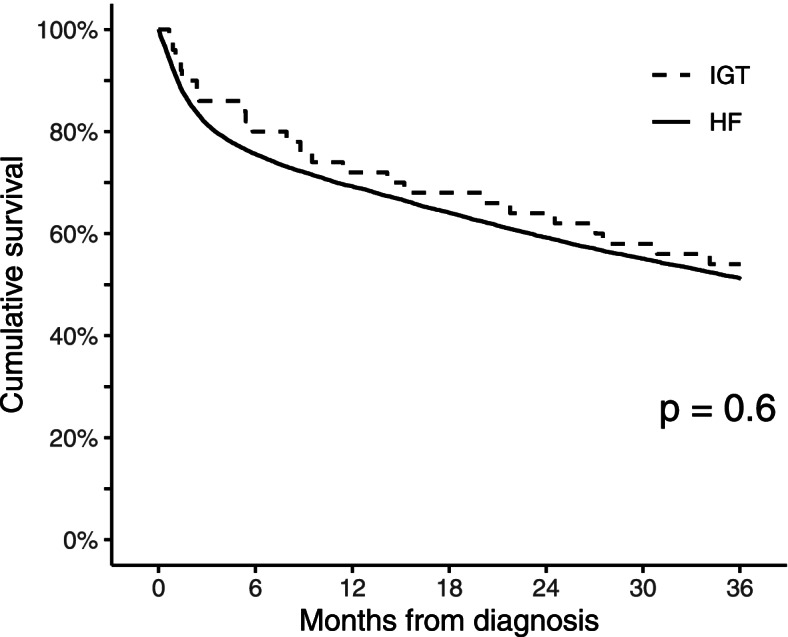
Three-year Kaplan–Meier survival curves and risk table for isolated greater trochanter (IGT) and conventional hip fracture (HF) patients. *P*-value shows the difference in three-year survival

The IGT patients were as likely to die as the patients with HF. The crude analysis showed almost matching three-year survival curves (Fig. 2). The IGT and HF patients’ mortality rates were comparable: 4% and 9% for one, 14% and 18% for three, 20% and 24% for six months, and 28% and 31% for one, 38% and 42% for two, and 46% and 49% for three years, respectively (Table 3). After adjusting for age, sex, and CCI, the IGT patients’ HR for the three-year follow-up was 0.9 [0.6; 1.3].

**Table 3:** Crude mortality rates of isolated greater trochanter and hip fracture patients

Time point	Total *n* = 11,541	Isolated greater trochanter fracture *n* = 50	Hip fracture *n* = 11,491	*P*-value
One month	988 (9)	2 (4)	986 (9)	0.2
Three months	2,132 (18)	7 (14)	2,125 (18)	0.2
Six months	2,813 (24)	10 (20)	2,803 (24)	0.4
One year	3,548 (31)	14 (28)	3,534 (31)	0.6
Two years	4,792 (42)	19 (38)	4,773 (42)	0.5
Three years	5,628 (49)	23 (46)	5,605 (49)	0.1

Mortality rates are presented as n (%). *P*-values are based on the Log-rank test

## Discussion

IGT may have a similar effect on older adults’ mortality as does HF, which is known to have a three- to four-times higher one-year mortality risk as compared to the general population [30]. Over one-fourth of the IGT patients died within one year, which is similar to the HF mortality rate [2–4]. Only two other studies have reported post-IGT mortality, and their findings are conflicting. One reported only one-month mortality that was relatively similar to that of HF [2–4, 11]. However, another study from the Republic of Korea reported that none of the 30 patients died within a year [9]. Several reasons may explain this discrepancy between the studies. The sample South Korean study was small. There also may be differences in population characteristics or fracture management since the South Korean HF mortality rate for one year is 11 percentage points lower than that of Estonia [31, 32]. Despite the controversy in the available literature, the present study confirmed the findings of Thurston and colleagues (2018), additionally providing mortality estimates for IGT patients from the first month onwards up to three years.

The similar mortality risks for IGT and HF patients may be explained by the close resemblance of their baseline characteristics, such as age, higher female ratio, and comorbid conditions. Older age and a higher female ratio are associated with underlying osteoporosis, which is also a risk factor for HF [33]. This is further supported by a study in which 87% of IGTs were caused by a minor indoor trauma [9]. All of the study’s patients had underlying osteoporosis [9]. While considering the similarities of the fracture populations, older IGT patients may also have underlying frailty, as approximately half of HF patients are frail [34–37]. Multiple components of frailty, including poor mobility, balance, and reduced muscle strength, are known risk factors for mortality, which could also explain the high mortality of IGT patients [30]. IGT can negatively affect the aforementioned components of frailty, as it causes persistent pain and hip abductor weakness for up to one year [38]. In summary, these findings indicate that IGT may have a similarly detrimental effect on older patients’ survival as conventional HF due to the similarities of these two fracture populations.

Despite the similarities of these fracture populations, the results of our study showed that IGT patients’ acute care was very brief, and most of them were excluded from post-acute care and physical and occupational therapy. The use of bone density scanning was negligible. The limited evidence-based guidelines may explain this since IGT is relatively rare [8, 10]. Taking also into account the similar baseline characteristics and mortality of these fracture populations, older IGT patients may also need similar holistic, multidisciplinary, ongoing, coordinated management strategies as do those with an HF [1, 39]. These strategies may include adequate pain control, controlling of delirium, nutrition, drug deprescribing and reconciliation, early rehabilitation, use of post-acute rehabilitation programs, patient involvement with their care programmes and decisions, secondary prevention of fragility fractures, falls assessment and management, orthopaedic specialist follow up [40]. Addressing the fracture only in treatment may lead to adverse outcomes due to the progression of other comorbidities. Thus, IGT in older patients may also signify an underlying need for broad-based medical attention. The most profound difference between these two populations is that treatment is surgical for the large majority of the HF patients and conservative for the IGT subpopulation. The nearly double 1-month mortality risk in the HF group is likely associated with the surgery. Even though early surgery is associated with better outcomes and lower mortality [41], it is a substantial risk for elderly HF patients. A recent systematic review reported that most studies recommend conservative treatment for IGT – only 0.6% of analysed patients were treated surgically [12]. The rationale for the treatment discrepancy arises from the substantially lower impact on the weight-bearing biomechanics of IGT.

Limited diagnostics may also explain the IGT patients’ relatively high mortality, as half of their diagnoses were based solely on conventional radiography, and this may not be sufficient to rule out occult intertrochanteric fractures. For this reason, studies predominantly recommend advanced imaging methods such as magnetic resonance imaging (MRI), computed tomography (CT), or bone scan with SPECT (after 48–72 h) for IGT diagnostics [7–10, 12–14, 38, 42–46]. Where advanced imaging methods are not available or where patients fail to progress, conventional radiography may be repeated [11]. Definitive fracture evaluation allows the identification of intertrochanteric extensions or complete fractures, which may require surgical fixation [9, 12, 15, 44]. The possibility that some of the IGT patients had an intertrochanteric extension, leading to a complete fracture, cannot be ruled out in this study. However, such patients would generally return to the hospital due to failure to progress or possible fracture displacement, so the probability of this scenario is low. This is further supported by the fact that only six of the deceased IGT patients had a diagnosis based on conventional radiography and lacked information about subsequent digital imaging and post-acute care.

The study has multiple strengths as it is based on validated, high-quality, nationwide, population-based, and nine-year-spanning data with complete, considerably long follow-ups for all patients. Nevertheless, limitations should be considered when interpreting the results of this study. The number of IGT patients was low, increasing the probability that the analyses were insufficiently powered for identifying a difference (risk of type II error). However, sufficient powering would be very hard, if not impossible, to achieve, making this limitation inevitable: IGT is rare, all relevant literature suffers from small sample sizes [8–10, 14, 46], and the survival curves of the studied populations almost matched (Fig. 2). Secondly, half of IGT diagnoses were based only on conventional radiography. Still, as discussed earlier, the possibility of having complete fractures due to an intertrochanteric extension is low while considering the data validation strategies. Finally, there is a possibility that some IGT patients were misdiagnosed as having an HF and received operative management.

## Conclusions

This is the first study comparing IGT and HF populations, their management, and long-term mortality, and it provides essential input for improving IGT management. Despite IGT being a relatively minor injury, it may impose a comparable risk on older patients’ survival, as does HF due to the close resemblance of the two fracture populations. Therefore, IGT in older patients may signify an underlying need for broad-based medical attention, ensuring need-based, ongoing, coordinated care. Lastly, these results need to be interpreted with some caution since the research on rare diseases inevitably includes a small number of patients, increasing the possibility of statistical analyses being underpowered.

## Data Availability

The dataset used and analysed during the current study is available from the corresponding author on reasonable request.
